# Diagnostic yield of endoscopic ultrasonography in patients with intermediate or high likelihood of choledocholithiasis: a retrospective study from one university-based endoscopy center

**DOI:** 10.1186/1471-230X-14-165

**Published:** 2014-09-26

**Authors:** Varayu Prachayakul, Pitulak Aswakul, Patommatat Bhunthumkomol, Morakod Deesomsak

**Affiliations:** Department of Internal Medicine, Siriraj GI Endoscopy Center, Siriraj Hospital, Division of Gastroenterology, Faculty of Medicine, Mahidol University, Bangkok, Thailand; Liver and Digestive Institute, Samitivej Sukhumvit Hospital, Bangkok, Thailand; Department of Medicine, Patommatat Bhunthumkomol, Gastrointestinal Unit, Thammasat University Hospital, Pathumthani, 12120 Thailand

**Keywords:** Endoscopic ultrasonography, Choledocholithiasis, Clinical likelihood, High likelihood, Intermediate likelihood

## Abstract

**Background:**

Diagnosis of choledocholithiasis requires clinical manifestations and imaging examination findings suggesting a stone in the common bile duct (CBD), but these factors are not highly sensitive or specific. The choledocholithiasis management algorithm proposed by the American Society for Gastrointestinal Endoscopy (ASGE) may not be appropriate for patients who fulfill the clinical criteria for a high likelihood of choledocholithiasis. Endoscopic ultrasonography (EUS) may replace endoscopic retrograde cholangiopancreatography (ERCP) for the detection of CBD stones in all patients. The aims of this study were to determine the diagnostic yield and optimal timing of EUS in patients with an intermediate or high likelihood of choledocholithiasis requiring therapeutic ERCP.

**Methods:**

Patients with suspected choledocholithiasis who underwent EUS between June 2009 and January 2012 were retrospectively reviewed. The patients were divided into two groups based on the likelihood of choledocholithiasis according to the clinical predictors described by the ASGE guidelines: an intermediate likelihood group and a high likelihood group. The demographic data, clinical manifestations at presentation, blood test results, EUS and ERCP findings, and clinical manifestations during the follow-up period were recorded and analyzed.

**Results:**

Ninety-three patients were enrolled in the study (52.7% in the intermediate likelihood group and 47.3% in the high likelihood group). CBD stones were detected in 22.44% of patients in the intermediate likelihood group and 38.63% of patients in the high likelihood group. EUS had a sensitivity of 100% and specificity of 80% for detection of CBD stones. An alkaline phosphatase level of >133 mg/dL (area under the curve, 0.576) was the only factor that was significantly associated with detection of CBD stones in patients who underwent EUS >7 days after the initial clinical presentation (odds ratio 4.87, *p* = 0.01).

**Conclusions:**

EUS is an accurate diagnostic tool for the detection of CBD stones, and can prevent the unnecessary use of ERCP. This study found that use of clinical criteria alone might not provide a good prediction of the presence of CBD stones, even in patients who fulfill the criteria for a high likelihood of choledocholithiasis.

## Background

Choledocholithiasis is a common condition, which occurs in 10–20% of patients with cholelithiasis, 7–14% of patients who have undergone cholecystectomy, and 18–33% of patients with acute biliary pancreatitis [1]. Management of patients with suspected choledocholithiasis requires confirmation of stones in the common bile duct (CBD).

Diagnosis of choledocholithiasis is based on clinical signs and symptoms, levels of serum markers of cholestasis, and imaging examination findings (transabdominal ultrasonography), but these factors are not highly sensitive or specific. Endoscopic retrograde cholangiopancreatography (ERCP) is the gold standard method for diagnosis and treatment of choledocholithiasis. However, as indiscriminate use of ERCP increases the risk of procedure-related complications [2], ERCP is almost exclusively reserved for therapeutic purposes, and choledocholithiasis is usually diagnosed using non-invasive methods.

The benefits of using endoscopic ultrasonography (EUS) for the detection of CBD stones were described by Amouyal et al. in 1989 [3]. EUS is less invasive than ERCP, and has excellent sensitivity and specificity for the detection of CBD stones [4, 5]. Considering the increasing availability of EUS in hospitals, and recent reports that EUS may have a higher diagnostic accuracy than magnetic resonance cholangiopancreatography for the detection of small CBD stones (<5 mm), EUS should be recommended for the detection of CBD stones instead of ERCP, to minimize the risk of procedure-related complications. According to the 2010 American Society for Gastrointestinal Endoscopy (ASGE) guidelines [6], which mainly cite the studies by Bakun et al. and Abboud et al. [7, 8], an EUS-guided management algorithm may be cost-effective for patients with an intermediate risk of choledocholithiasis, while an ERCP-guided approach may be more economic in patients with a high risk of choledocholithiasis. However, we experienced several patients at our institution who fulfilled the criteria for a high likelihood of choledocholithiasis and underwent ERCP, in whom no stones were detected. Ang et al. [9] reported that CBD stones were detected in only 29.6% of the 112 patients in their study who fulfilled the criteria for a high likelihood of CBD stones, which is lower than previously reported rates [9–13]. We postulate that discrepancies between the likelihood of choledocholithiasis and the detection of CBD stones on EUS may be related to the length of time between the initial clinical presentation and the EUS examination, and that ERCP may not always be more appropriate than EUS in patients who fulfill the criteria for a high likelihood of choledocholithiasis. The aims of this study were to determine the diagnostic yield and optimal timing of EUS in patients with an intermediate or high likelihood of choledocholithiasis requiring therapeutic ERCP, and to identify the factors associated with detection of CBD stones in our endoscopy clinics.

## Methods

### Study population

This study was approved by the institutional review board of Siriraj Hospital. Patients who presented with clinical manifestations suggestive of choledocholithiasis, who had inconclusive findings on initial imaging examinations and subsequently underwent EUS between June 2009 and January 2012, were included in the study. The medical records were retrospectively reviewed and the demographic data, clinical manifestations at presentation, blood test results, imaging examination findings, EUS and ERCP findings, and clinical manifestations during the follow-up period were recorded and analyzed.

**Figure 1:**
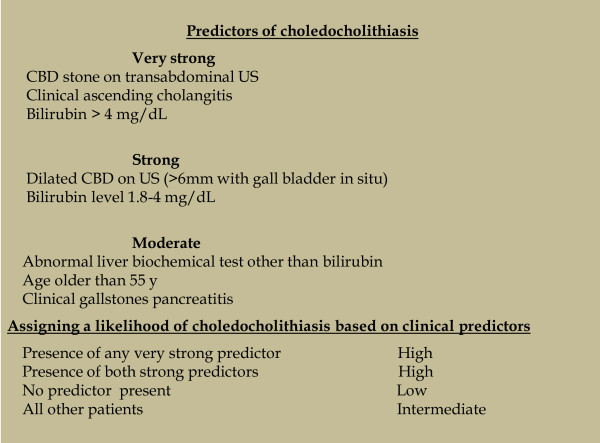
**Clinical predictors of choledocholithiasis.** *CBD* common bile duct, *US* ultrasonography.

Patients were included in the study if they had at least two of the following clinical manifestations of choledocholithiasis: right upper abdominal or epigastric pain, jaundice, fever, unexplained derangement of liver function tests, or abnormal findings on imaging examinations of the hepatobiliary tract such as a dilated CBD (≥7 mm with an intact gallbladder or ≥10 mm after cholecystectomy) or a suspected CBD stone. The exclusion criteria were: age <18 years, pregnancy, and inability to provide informed consent. The patients were divided into two groups based on based on the likelihood of choledocholithiasis according to the clinical predictors described by the ASGE guidelines: an intermediate likelihood group and a high likelihood group (Figure 1) [6].

### EUS examinations

Patients underwent EUS using a GF-UE160 radial echoendoscope (Olympus Corporation, Tokyo, Japan). EUS was performed by a dedicated endoscopist who was experienced in performing EUS at our university-based hospital, where more than 400 EUS procedures are performed annually. EUS was performed under conscious sedation administered by an anesthesiologist using midazolam, fentanyl, or propofol. The EUS findings were considered positive if hyperechoic foci were detected in the CBD, with or without associated acoustic shadows or biliary sludge. If the EUS findings were positive, patients underwent ERCP to remove the stones using a TJF-160VR duodenoscope (Olympus Corporation), by the same endoscopist as part of the same procedure. If EUS did not detect CBD stones, patients were followed up for at least 6 months after the index EUS procedure. If patients were lost to follow-up before the end of the 6-month follow-up period, their status was assessed by telephone. Four patients with negative EUS findings underwent ERCP because the consulting surgeon required a definitive diagnosis prior to performing laparoscopic cholecystectomy. Positive EUS findings were only considered truly positive if stones or sludge were visualized by ERCP. Patients with truly positive findings underwent sphincterotomy followed by basket or balloon sweeping of the bile duct. Negative EUS findings were considered truly negative if no stones or sludge were found during ERCP with balloon sweeping of the bile duct. A negative outcome associated with an EUS-based false-negative diagnosis was defined as detection of CBD stones during the follow-up period associated with pain thought to be of biliary origin, cholangitis, or acute pancreatitis. Clinical, blood test, and endoscopic findings were compared between patients with and without detection of CBD stones.

### Statistical analysis

Descriptive data are presented as mean and standard deviation (SD). Factors associated with CBD stones were assessed by univariate analyses using the χ^2^-test, and by multivariate logistic regression analysis with calculation of odds ratios and 95% confidence intervals (CIs). The sensitivity, specificity, negative predictive value, and positive predictive value of EUS for detection of CBD stones were calculated, using the ERCP findings as the gold standard. All statistical analyses were performed using SPSS software version 18.0.

## Results

Ninety-three patients were included in the study, out of a total of 914 patients who underwent ERCP for CBD stone removal between June 2009 and January 2012. The mean (±SD) age of the patients was 61.0 (±15.6) years (range, 26–85 years), and 47 (50.5%) were men. The mean (±SD) time between the initial clinical presentation and the EUS examination was 37.6 (±56.6) days (range, 1–420 days). The most common clinical manifestations were abdominal pain, jaundice, and fever, which occurred in 74.2%, 29.0%, and 23.7% of patients, respectively. Leukocytosis was found in 25.8% of patients. The bilirubin level was abnormal in 53.2% of patients, and the mean (±SD) total bilirubin level was 2.9 (±4.4) mg/dL (range, 0.2–34.8 mg/dL). Abnormal liver enzyme levels were found in 71.2% of patients. The mean (±SD) serum glutamic-oxaloacetic transaminase level was 169.9 (±206.9) U/L (range, 10–936 U/L), the mean (±SD) serum glutamic-pyruvic transaminase level was 158.3 (±203.2) U/L (range, 7–1376 U/L), and the mean (±SD) alkaline phosphatase level was 174.3 (±156.4) U/mL (range, 36–885 U/mL). According to the clinical classification criteria described previously [6], 52.7% of the patients had an intermediate likelihood of choledocholithiasis and 47.3% had a high likelihood choledocholithiasis.

EUS found CBD stones in 29 of the 93 patients (31.2%). Thirty-three patients underwent ERCP on the same day as their EUS examination, including 29 with CBD stones detected on EUS (28 truly positive) and 4 without CBD stones detected on EUS (all truly negative). EUS therefore had a sensitivity of 100% and specificity of 80% for detection of CBD stones, with a positive predictive value of 96.55% and a negative predictive value of 100%. Patients who did not have CBD stones detected on EUS were followed up. The mean (±SD) follow-up period was 10.2 (±7.9) months (range, 1–41 months). Thirty-four patients (36.6%) were lost to follow-up within 6 months. All the patients lost to follow-up were contacted by telephone, and none of them experienced any abdominal pain suspicious of biliary colic or underwent ERCP for stone removal at another hospital.

Table 1 shows the EUS and ERCP findings. CBD stones were detected in 22.44% of patients with an intermediate likelihood of choledocholithiasis and 38.63% of patients with a high likelihood of choledocholithiasis.

**Table 1:** **Detection of CBD stones by EUS and ERCP**

	CBD stone detected by EUS n (%)	CBD stone detected by ERCP n (%)
Intermediate likelihood (n = 49)	11 (22.44%)	11 (22.44%)
High likelihood (n = 44)	18 (40.90%)	17 (38.63%)

*CBD* common bile duct, *EUS* endoscopic ultrasonography, *ERCP* endoscopic retrograde cholangiopancreatography.

Univariate analyses of the factors potentially associated with choledocholithiasis are shown in Table 2. An elevated alkaline phosphatase level and EUS examination within 7 days of the initial clinical presentation were associated with detection of CBD stones.

**Table 2:** **Univariate analyses of factors potentially associated with detection of CBD stones**

	No CBD stone detected (n = 65)	CBD stone detected (n = 28)	***p***value
Mean (±SD) age (years)	58.85 (±16.09)	65.26 (±13.76)	0.06
Mean (±SD) alkaline phosphatase level (U/mL)	147.40 (±127.55)	226.30 (±192.64)	0.02*
Mean (±SD) total bilirubin level (mg/dL)	3.19 (±5.20)	2.57 (±2.33)	0.54
Mean (±SD) SGOT level (U/L)	215.13 (±176.86)	215.13 (±250.75)	0.13
Mean (±SD) SGPT level (U/L)	137.07 (±210.94)	198.65 (±184.04)	0.17
**Clinical classification**			0.06
Intermediate likelihood (n = 49)	38	11	
High likelihood (n = 44)	27	17	
Dilated CBD on transabdominal			
ultrasonography (n = 26)	5	21	0.48
Mean (±SD) time interval (days)	38.95 (±62.77)	33.23 (±42.24)	0.64
EUS performed within 7 days (n)	14	14	0.02*

*CBD* common bile duct, *EUS* endoscopic ultrasonography, *ERCP* endoscopic retrograde cholangiopancreatography, *SGOT* serum glutamic-oxaloacetic transaminase, *SGPT* serum glutamic-pyruvic transaminase.

Subgroup analysis was performed to compare patients who underwent EUS within 7 days of the initial clinical presentation and patients who underwent EUS more than 7 days after the initial clinical presentation. An alkaline phosphatase level of >133 mg/dL (area under the curve, 0.576) was the only factor associated with detection of CBD stones in patients who underwent EUS more than 7 days after the initial clinical presentation (odds ratio: 4.87, *p* = 0.01) (Table 3). Additional analyses of individual or combined choledocholithiasis predictors that are classified as “strong” or “very strong” in the ASGE guidelines [6] did not show significant associations between these factors and the detection of CBD stones (data not shown).

**Table 3:** **Detection of CBD stones in patients with an alkaline phosphatase level >133 mg/dL, who underwent EUS ≤7 days and >7 days after the initial clinical presentation**

	No CBD stone detected (n = 65)	CBD stone detected (n = 28)	***p***value
Alkaline phosphatase level >133 mg/dL and EUS ≤7 days after presentation (n = 17)	7	10	0.29
Alkaline phosphatase level >133 mg/dL and EUS >7 days after presentation (n = 25)	14	11	0.01*

*CBD* common bile duct, *EUS* endoscopic ultrasonography.

## Discussion

The reported sensitivity and specificity of non-invasive imaging examinations for the detection of CBD stones are 25–58% and 68–91%, respectively, for transabdominal ultrasonography; and 87% (95% CI: 84–90) and 97% (95% CI: 95–98), respectively, for computed tomography [14]. Magnetic resonance cholangiopancreatography has a sensitivity of approximately 91% (95% CI: 73–97) for detection of CBD stones, depending on the stone size as follows: 67–100% for stones >10 mm, 89–94% for stones 6–10 mm, and 33–71% for stones <6 mm [14]. The optimal initial non-operative investigation for the detection of CBD stones is therefore still unclear.

Although ERCP is the gold standard method for detection of CBD stones, with a reported sensitivity of 90% and specificity of 98%, indiscriminate use of ERCP results in an increased rate of associated complications. ERCP is therefore almost exclusively used for therapeutic purposes, with non-invasive tests used for the detection of CBD stones. EUS is a new, less invasive imaging technique that has shown very good sensitivity (95%) and specificity (98%) for the detection of CBD stones [4, 5]. In this study, EUS detected CBD stones in all the patients who were truly positive for CBD stones, with only one false-positive case. Retrospective review of the EUS images from the false-positive case showed a suspected echo artifact. EUS should be the imaging examination of choice for the detection of CBD stones, because of its high sensitivity and specificity. EUS is as effective as ERCP for detecting CBD stones, and has fewer procedural risks and complications. However, EUS also has some disadvantages, including the high cost, the need for expertise in the procedure, and the lack of availability in some hospitals.

The present study included almost equal numbers of patients with intermediate and high likelihood of choledocholithiasis. CBD stones were detected in 22.44% of patients in the intermediate likelihood group and 38.63% of patients in the high likelihood group. These results differ from previous studies, which reported detection of CBD stones in up to 40% of patients in the intermediate likelihood group and 60% of patients in the high likelihood group [10–15]. We postulate that the lower rate detection of CBD stones in our study may reflect spontaneous passage of the stones, which is reported to occur at a rate of 20% per week [16], and would be affected by the time between the initial clinical presentation and the EUS examination. Our findings therefore support the potential cost-effectiveness of EUS for patients with an intermediate likelihood of choledocholithiasis suggested by the ASGE algorithm, and the performance of EUS-guided ERCP. However, routine ERCP may not be appropriate for all patients with a high likelihood of choledocholithiasis.

In the past decade, many studies have demonstrated that EUS is as accurate as ERCP for the detection of CBD stones, and that EUS is associated with fewer complications than ERCP [17–22]. However, EUS is not available in all hospitals, especially in developing countries. Currently, EUS is only available in large tertiary hospitals, which may result in delay between the initial clinical presentation and the EUS examination. In the current study, patients who underwent EUS within 7 days of the initial presentation were more likely to have positive findings than patients who underwent EUS more than 7 days after the initial clinical presentation. Our analyses show that EUS more than 7 days after the initial clinical presentation and an elevated alkaline phosphatase level were associated with detection of CBD stones, especially when the alkaline phosphatase level was >133 mg/dL.

This study was limited by its retrospective design and the relatively small number of patients. The results are therefore only applicable to similar healthcare situations. Further prospective studies should be conducted to confirm the findings of this study.

## Conclusions

EUS is an accurate diagnostic tool for the detection of CBD stones, and can reduce the unnecessary use of ERCP. This study demonstrated that use of clinical criteria alone might not provide good predictions regarding the presence of CBD stones in all patients, even those who fulfill the criteria for a high likelihood of choledocholithiasis. EUS examination should be considered for patients with a high likelihood of choledocholithiasis, especially those who cannot undergo ERCP within 7 days of the initial clinical presentation or who have a high risk of ERCP-related complications, to minimize unnecessary ERCP procedures.

## Abbreviations

ASGE: American society for gastrointestinal endoscopy
CBD: Common bile duct
CI: Confidence interval
ERCP: Endoscopic retrograde cholangiopancreatography
EUS: Endoscopic ultrasonography
SD: Standard deviation.
